# Agglomeration State of Titanium-Dioxide (TiO_2_) Nanomaterials Influences the Dose Deposition and Cytotoxic Responses in Human Bronchial Epithelial Cells at the Air-Liquid Interface

**DOI:** 10.3390/nano11123226

**Published:** 2021-11-27

**Authors:** Sivakumar Murugadoss, Sonja Mülhopt, Silvia Diabaté, Manosij Ghosh, Hanns-Rudolf Paur, Dieter Stapf, Carsten Weiss, Peter H. Hoet

**Affiliations:** 1 Laboratory of Toxicology, Unit of Environment and Health, Department of Public Health and Primary Care, KU Leuven, 3000 Leuven, Belgium; manosij.ghosh@kuleuven.be (M.G.); peter.hoet@kuleuven.be (P.H.H.); 2 Institute for Technical Chemistry, Karlsruhe Institute of Technology, 76021 Karlsruhe, Germany; hanns@dr-paur.net (H.-R.P.); dieter.stapf@kit.edu (D.S.); 3 Institute of Biological and Chemical Systems—Biological Information Processing, Karlsruhe Institute of Technology, 76021 Karlsruhe, Germany; sildia76344@gmx.de (S.D.); carsten.weiss@kit.edu (C.W.)

**Keywords:** nanomaterials, titanium dioxide, agglomerates, air-liquid interface, pulmonary toxicity

## Abstract

Extensive production and use of nanomaterials (NMs), such as titanium dioxide (TiO_2_), raises concern regarding their potential adverse effects to humans. While considerable efforts have been made to assess the safety of TiO_2_ NMs using in vitro and in vivo studies, results obtained to date are unreliable, possibly due to the dynamic agglomeration behavior of TiO_2_ NMs. Moreover, agglomerates are of prime importance in occupational exposure scenarios, but their toxicological relevance remains poorly understood. Therefore, the aim of this study was to investigate the potential pulmonary effects induced by TiO_2_ agglomerates of different sizes at the air–liquid interface (ALI), which is more realistic in terms of inhalation exposure, and compare it to results previously obtained under submerged conditions. A nano-TiO_2_ (17 nm) and a non-nano TiO_2_ (117 nm) was selected for this study. Stable stock dispersions of small agglomerates and their respective larger counterparts of each TiO_2_ particles were prepared, and human bronchial epithelial (HBE) cells were exposed to different doses of aerosolized TiO_2_ agglomerates at the ALI. At the end of 4h exposure, cytotoxicity, glutathione depletion, and DNA damage were evaluated. Our results indicate that dose deposition and the toxic potential in HBE cells are influenced by agglomeration and exposure via the ALI induces different cellular responses than in submerged systems. We conclude that the agglomeration state is crucial in the assessment of pulmonary effects of NMs.

## 1. Introduction

Nanotechnology is ubiquitous, brings novel advancements in all aspects of human life on a daily basis, and has a wide variety of applications, such as in consumer goods, electronics, communication, environmental treatments and remediations, agriculture, nanomedicine, water purification, textiles, aerospace industry, and efficient energy sources, among many others. The field of nanotechnology is one of the fastest expanding markets in the world and its global value is expected to exceed the USD 125 billion mark by 2024 [1].

Nanomaterials (NMs) are generally defined as a material with at least one dimension in the nanoscale (1–100 nm) range [2]. While NMs are abundant in nature and produced by various sources, such as forest fires and volcanic eruptions, they are also intentionally manufactured by nanotechnologies on a global scale for industrial and commercial purposes. EU recommended a definition for NM solely for regulatory purpose, which states that “natural, incidental or manufactured material containing particles, in an unbound state or as an aggregate or as an agglomerate and where, for 50% or more of the particles in the number size distribution, one or more external dimensions is in the size range 1 nm–100 nm” [3].

Among the manufactured NMs, titanium dioxide (TiO_2_) is one of the widely used NMs in commercial applications and approximately four million tons of TiO_2_ are produced annually worldwide [4,5,6]. Commercial TiO_2_ NMs come in different crystalline forms such as anatase and rutile. As TiO_2_ NMs reflect UV light, they are widely used in cosmetics and in paints as a UV filter [5] as well as in plastics [7]. TiO_2_ NMs are also extensively used as food colourant (food additive E171) [8]. Due to their light dependent properties, TiO_2_ NMs are being studied for potential medical and bio-medical applications such as antibacterial activity, biosensing, drug delivery, and implant applications [9,10]. The production of TiO_2_ NMs is expected to expand continuously due to their potential in the energy sector and environmental based applications [11]. This clearly indicates that there is a potential for human exposure, particularly inhalation, as this is the major route of exposure to TiO_2_ NMs in occupational settings and raises concerns about their safety and adverse pulmonary effects [12].

Toxicological evaluations of TiO_2_ NMs are often performed using in vivo models such as mice and rats. Short and long term exposure to TiO_2_ NMs via inhalation induced pulmonary inflammation, fibrosis and tumours [6,13,14,15]. A significant increase in cytotoxicity, inflammation, oxidative stress, and DNA damage was observed in mice exposed to high doses (10 mg/kg [16] and ~4 mg/kg [17]) of TiO_2_ NMs. The studied endpoints are major key events identified to play essential roles in fibrosis and tumour development [14,18].

In vitro models are often employed as a first screening method, to unveil the mechanisms involved in the induction of adverse effects, and to prioritize NMs for further animal testing. Traditionally, submerged in vitro cell cultures are widely used to assess the adverse effects of NMs with a particular focus on the production of reactive oxygen species, which can be generated specially in case of TiO_2_ NMs [19]. Submerged exposure to TiO_2_ NMs induced cytotoxicity, oxidative stress, pro-inflammatory responses, and genotoxicity in lung derived immortalized cell lines [6]. In submerged exposure systems, the cells are covered with culture media to which NMs are added. The biomolecules present in the culture media can adsorb to the surface of the NMs to form a protein corona [20,21]. Such changes to the surface can potentially prevent the adverse effects of NMs [22], affect the physico-chemical properties relevant for toxicological assessment (size, surface area, surface composition, surface charge, and agglomeration, etc.) [23], and also effective density [24], an important parameter that determines the sedimentation of NMs. However, these modifications of NMs in cell culture medium often do not reflect the conditions upon inhalation in real life situations.

Recently, exposure at the air liquid interface (ALI) has been evolving as a potential alternative to conventional submerged in vitro exposure systems. At the ALI, cells grown on transwell plates are directly exposed to aerosolized particles and gases, which better reflects the exposure in vivo via inhalation [25,26,27,28]. Previously, we have developed, validated, and used a fully integrated ALI exposure system for the assessment of toxicological effects of various NMs and aerosols [29,30,31,32,33].

It is well known that the physicochemical properties of NMs influence their toxicity [34]. Among all the properties, the influence of agglomeration on the toxicity of NMs is less well studied and poorly understood. In our previous study, we assessed the influence of TiO_2_ NM agglomeration on (cyto) toxicity and biological responses [35] using a human bronchial epithelial (HBE) cell line. However, the entire study was carried out in submerged exposure conditions. While there is only a limited number of toxicological investigations addressing adverse effects of TiO_2_ NMs using ALI exposure systems [31,36,37], the impact of agglomeration has not been researched. Here, we prepared TiO_2_ NM agglomerates of different sizes and performed toxicological studies employing ALI exposure. The aim of the present work was to investigate the cytotoxicity and biological responses in HBE cells after ALI exposure to different doses of TiO_2_ agglomerates of different sizes and compare the results to those previously obtained under submerged conditions [35].

## 2. Materials and Methods

### 2.1. Preparation of Dispersions and Characterization of TiO_2_ NMs

Two TiO_2_ NMs (representative test materials) of different primary sizes were kindly provided by the European Commission’s Joint Research Centre (JRC, Ispra, Italy). Mean primary size of TiO_2_-JRCNM10202a was determined as 17 nm and TiO_2_-JRCNM10200a as 117 nm. Therefore, the two NMs are indicated as 17 nm and 117 nm sized TiO_2_ in the text. Both TiO_2_ NMs are pristine and anatase in nature. Detailed physicochemical characterization of these NMs were provided in a previously published JRC report [38].

Detailed information on the development of the dispersion protocol to obtain two different agglomeration states (small and large agglomerates) of both TiO_2_ NMs were published elsewhere [17,35]. Briefly, to obtain agglomerates of different sizes, particles were dispersed in different pH conditions (2 and 7), the dispersions were probe sonicated (7056 J) and stabilized with 1% bovine serum albumin (BSA). After stabilization, the suspensions at pH 2 were readjusted to pH 7–7.5 by slowly adding sodium hydroxide solution (NaOH). While the original dispersion protocol was developed to prepare 10 mL of stock dispersions and intended for submerged exposure, for ALI exposure, the quantity was scaled up to 120 mL to provide sufficient quantity of dispersions for the aerosolization of TiO_2_ agglomerates during the 4 h exposure period. Each dispersion was freshly prepared before each exposure. Table 1 shows the nomenclature of different dispersions.

### 2.2. Cell Culture Maintenance

The human bronchial epithelial cell line (16HBE14o- or HBE) was kindly provided by Dr. Gruenert (University of California, San Francisco, CA, USA). HBE cells were cultured in DMEM/F12 supplemented with 5% fetal bovine serum (FBS), 1% penicillin-streptomycin (P-S) (100 U/mL), 1% L-glutamine (2 mM) and 1% fungizone (2.5 g/mL). All cell culture supplements were purchased from Invitrogen (Merelbeke, Belgium) unless otherwise stated. Cells were cultured in T75 flasks at 37 °C in a 100% humidified air containing 5% CO_2_. Medium was changed every 2 or 3 days and cells were passaged every week (7 days). Cells from passage 4 to 8 were used for the experiments.

### 2.3. Air–Liquid Interface Exposure

For ALI exposure, 3.5 × 10^5^ HBE cells/mL were seeded on the apical side of a 6-well transwell plate (Corning Costar Transwell insert membranes type 3450, culture area 4.67 cm², pore size 0.4 µm, cat no 10619141, Fischer scientific, Schwerte, Germany) with 1.5 mL of cell culture medium on the basolateral side and incubated overnight at 5% CO_2_ and at 37 °C. Before ALI exposure, the apical and the basolateral media were removed. Then, cell culture medium was added into the basolateral compartment and the apical side was left uncovered (no medium). Uncovered cells were exposed to “clean air” (humidified synthetic air as negative control) or in parallel to airborne TiO_2_ agglomerates at low dose without electrostatic deposition and at different levels of electrostatic deposition (400 V, 800 V, and 1200 V) for 4h to facilitate dose–response evaluation of different biological endpoints. After exposure, the medium at the basal side of the transwell inserts was collected for LDH analysis. 

### 2.4. Aerosol Generation and Characterization 

**Figure 1:**
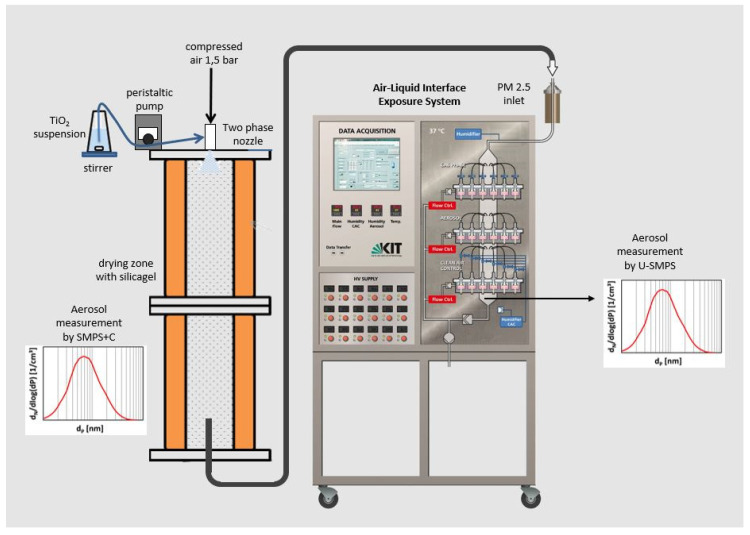
Experimental setup: generation of airborne TiO_2_ agglomerates in dry air according to VDI guideline 3491 and exposure of human lung cells in the Air–Liquid-Interface Exposure System with accompanying measurement of particle size distribution using Scanning Mobility Particle Sizer (SMPS) at the dry stage in the reactor as well as for the aerosol inside the exposure system.

Figure 1 shows the layout of the ALI exposure system. For TiO_2_ aerosol generation, a setup according to the VDI guideline 3491 (Technical Division Environmental Measurement Technologies, 2016) was used. The TiO_2_ dispersions, continuously stirred during the experiment, were sprayed in a drying reactor with a silica gel fill along the walls using a two-substance nozzle (model 970, Düsen-Schlick GmbH, Untersiemau/Coburg, Germany). The dry TiO_2_ aerosol was regularly characterized for the number size distribution in the drying reactor using a Scanning Mobility Particle Sizer SMPS + C (Grimm Aerosol GmbH, Ainring, Germany) and directed to the ALI exposure system, as described by Mülhopt et al. [30]. In the conditioning reactor of the ALI exposure system, the TiO_2_ aerosol is tempered to 37 °C and humidified to 85% r. h., and then sampling streams are directed to the single exposure chambers containing the cell cultures using an exposure flow rate of 100 mL/min. For describing the aerosol state as exposed to the cell cultures, the number size distribution was also measured by a Scanning Mobility Particle Sizer U-SMPS (Palas GmbH, Karlsruhe, Germany) sampled in the aerosol conditioning reactor. Every 5 min a scan was performed; means and standard deviation were calculated from all scans of an experiment in each channel and corrected regarding sampling losses according to Asbach et al. [39].

### 2.5. Determination of the Deposited Dose

The deposited cell culture surface dose is reflected by the deposited fraction of the TiO_2_ agglomerates exposed as aerosol towards the cells and not easy to determine. For this reason, three different methods were applied: the online monitoring of mass dose using the quartz crystal microbalance QCM (Vitrocell Systems GmbH, Waldkirch, Germany) [29], the image analysis of exposed TEM grids as presented in [40] and the calculation from the SMPS measured number size distribution as shown in [30]. The effective density of all TiO_2_ agglomerates were used as determined and reported in [35], and did not differ much between the dispersions ((17nm-SA: 1.55 g/cm^3^), (17nm-LA: 1.48 g/cm^3^), (117nm-SA: 1.78 g/cm^3^), and (17nm-LA: 1.78 g/cm^3^)).

### 2.6. Metabolic Activity

Metabolic activity was evaluated as a measure of cell viability using the WST-1 assay (Merck, Overijse, Belgium). At the end of ALI exposure, cells were washed with HBSS and incubated with 500 µL of WST1 reagent (diluted in HBSS at the ratio of 1:10) for 45 min. At the end of incubation, 100 µL was transferred to a 96 well plate and optical density was recorded at 450 nm. Sample OD values were subtracted from blank OD values and results were expressed as percentage of negative control cells. Cells exposed to clean air were treated as negative control and Triton X-100 (0.1%) lysed cells were treated as positive control (data not shown). 

### 2.7. Membrane Integrity

Lactate dehydrogenase (LDH) activity in the cell culture supernatant was measured as an indicator of membrane damage. Briefly, 100 µL of cell culture medium collected at the basal side at the end of ALI exposure were transferred to a 96 well plate and incubated with LDH mixture (prepared as indicated in the manufacturer’s protocol, Sigma-Aldrich, Taufkirchen, Germany, cat no 11644793001) and the optical density (OD) was recorded at 490 nm. Sample OD values were subtracted from blank OD values and results were expressed as percentage of Triton X-100 (0.1%) lysed cells. Cells exposed to clean air were treated as negative control.

### 2.8. Total Glutathione Measurements

Reduced glutathione (GSH) depletion was measured as an indicator of oxidative stress induction. Briefly, exposed cells were scraped, transferred into Eppendorf tubes and centrifuged at 150× *g* for 5 min. Then, the supernatants were discarded and cells were resuspended in 1 mL of phosphate-buffered saline (PBS). After centrifugation, PBS was removed and 450 µL of 10 mM hydrochloric acid (HCL) was added to each tube. Cell lysis was performed by the freeze thawing procedure (15 min freezing, 15 min thawing for two times) and immediately protein content analysis (by BCA assay) was performed using 10 µL of the cell lysate. Then, the lysate was resuspended in 6.5% 5-sulfosalicylic acid (SSA), incubated on ice for 10 min and centrifuged at 20,800× *g* (14,000 rpm) for 10 min at 4 °C to precipitate the proteins. The supernatants were stored at −80 °C for later GSH determination. GSH was measured using a glutathione detection kit (Enzo life sciences, Brussels, Belgium). Cells exposed to clean air were treated as negative control.

### 2.9. DNA Damage

Briefly, at the end of the ALI exposure, the cells were detached with trypsin, centrifuged at 250× *g* for 5 min, suspended in the storage buffer, composed of sucrose 85.5 g/L, dimethyl sulfoxide (DMSO) 50 mL/L prepared in citrate buffer (11.8 g/L), pH 7.6, and immediately frozen at −80 °C. DNA strand breaks were measured using the alkaline comet assay kit (Trevigen, C.No.4250–050-K, Gaithersburg, MD, USA) according to the manufacturer’s protocol. Fifty cells per gel were measured. Cells exposed to clean air were treated as negative control and cells treated with methyl methane sulfonate (Merck, Overijse, Belgium; 100 μM for 1h) served as positive control (data not shown). Results were expressed as percentage of DNA in the tail. NMs can interfere with the comet assay [41]. To evaluate this, we mixed TiO_2_ NMs with clean air exposed cells (negative control) and cells treated with MMS (positive control), performed the comet assay, and compared the results with negative and positive controls prepared without TiO_2_ NMs. The results indicated that the TiO_2_ NMs at high concentrations (100 µg/mL) did not interfere with the assay.

### 2.10. Statistical Analysis

Two or three independent experiments were performed with six replicates each and data was presented as mean ± standard deviation (SD). Using GraphPad prism 7.04 for windows, GraphPad Software (7.04, La Jolla, CA, USA), www.graphpad.com (accssed on 24 November 2021), the results were analysed with one-way ANOVA followed by a Dunnett’s multiple comparison test to determine the significance of differences compared with control.

## 3. Results

### 3.1. Size Characterization in Stock Suspensions

We obtained four agglomerate dispersions from two TiO_2_ NMs of different sizes. Detailed information on the physicochemical characterization and methods used to characterize the agglomerates in stock dispersions were published elsewhere [17,35]. Using a standardized TEM technique in our previous study [42], we measured the size of several thousand agglomerates in each dispersion. The TEM based determination of the diameter (median feret min) indicated that the size of 17 nm sized TiO_2_ NMs in their least agglomerated form (indicated as 17 nm-SA) was 33 nm, while it was 120 nm for the strongly agglomerated condition (17 nm-LA). The sizes of small (117 nm-SA) and large agglomerates (117 nm-LA) of 117 nm sized TiO_2_ NMs were 148 and 309 nm, respectively (see Figure S1 and Table S1). In summary, at low pH agglomeration of TiO_2_ NMs was modest whereas at neutral pH strong agglomeration of the small (17 nm) and less pronounced for the larger (117 nm) TiO_2_ NM is observed [17,35].

### 3.2. Aerosol Characterization and Determination of Deposited Dose 

**Figure 2:**
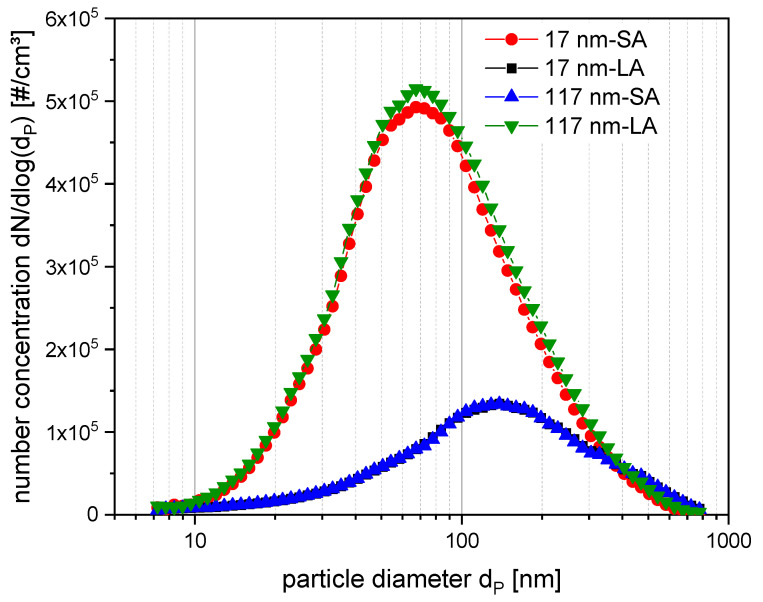
Particle size distributions of TiO_2_ agglomerates measured by Scanning Mobility Particle Sizer U-SMPS in the range of 8 to 800 nm. Each curve shows the means of number size distributions in dependence of particle type and agglomeration state. Red circles: 17 nm-SA; black squares: 17 nm-LA; blue triangles: 117 nm-SA; and green inverted triangles: 117 nm-LA.

The particle number size distributions (Figure 2) showed nearly the same characteristics for 17 nm-SA and 117 nm-LA with modal values x_M_ of 72 and 71 nm, respectively. The other titania 17 nm-LA and 117 nm-SA were also nearly identical with a size of x_M_ = 144 and 139 nm, respectively. All particle number size distributions have a typical geometric standard deviation σ_geo_ in the range of 2. These results show a similar trend as our previously reported TEM sizes (Supplementary Material Figure S1, [35]) for the different agglomerates in the stock solutions except for 117 nm-LA. Comparing SMPS and primary TEM data from stock solutions, the aerosol processing may cause differences for the size determination of 117 nm-LA agglomerates as the aerosol is characterized with the SMPS under nearly dry conditions, whereas for submerged exposure and subsequent TEM analysis, the water content and media components might increase the size of agglomerates. In addition, the SMPS measurements only cover the range of 10 to 800 nm and neglect possible larger agglomerates.

In Table 2, the summary of all measurements regarding TiO_2_ aerosol characteristics is listed. The mass concentration is calculated from the number size distribution of the SMPS measurements. The QCM was operated without electrostatic deposition delivering the diffusional doses as listed. Image evaluation of exposed TEM grids provides deposited surface doses for the 17 nm-LA and 117 nm-SA, both corresponding very well with the QCM data in the case of diffusional deposition (Supplementary Material, Figure S2). The enhanced doses for electrostatic deposited agglomerates were evaluated for the 17 nm-LA and 117 nm-SA from the exposed TEM grids. For these two types of agglomerates, the dose enhancement factors were determined. In case of 117 nm-SA, deposition enhancement factors of 5 for 400 V, 12 for 800 V, and 13 for 1200 V were calculated. Similarly, in case of 17 nm-LA deposition enhancement factors of 4 for 400 V, 9 for 800 V, and 9 for 1200 V are derived. For both types of agglomerates, the relative increase in deposition becomes less with the increase of the electrostatic field strength. This saturation behavior has been also shown earlier [40] and occurs when all charged agglomerates are deposited. In the TEM images of 17 nm-SA and 117 nm-LA individual particles could not be unambiguously identified due to a strong background signal, (Supplementary Material Figure S2), the doses listed in the summary table were calculated on the basis of the QCM data (measured at 0 V) multiplied by the enhancement factors derived at the different voltages for the corresponding particle types 17 nm-LA and 117 nm-SA. 

### 3.3. Cytotoxicity: Effect on Metabolic Activity and LDH Release

**Figure 3:**
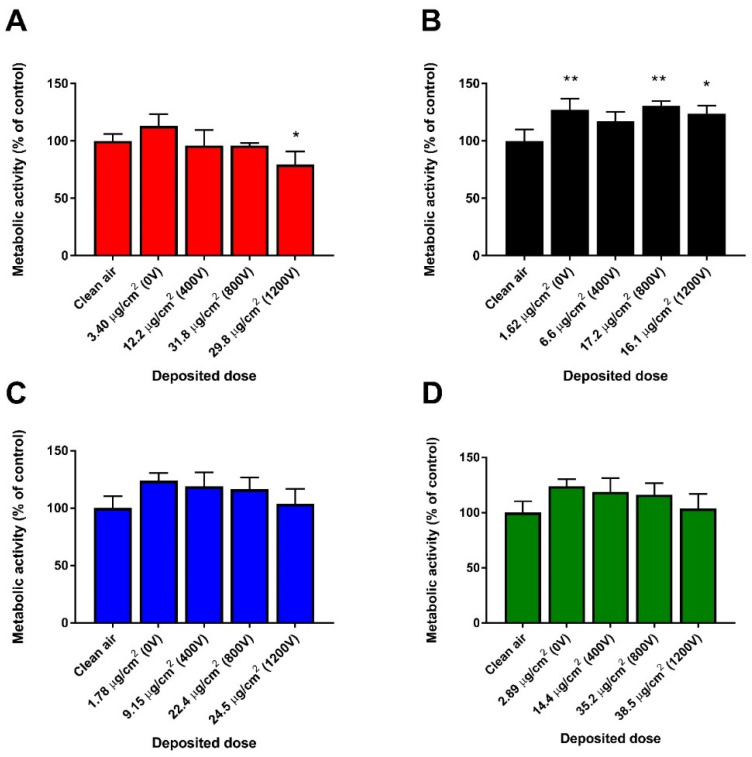
Effect on metabolic activity of HBE cells after 4 h exposure to TiO_2_ agglomerates at the ALI. 17 nm-SA (**A**), 17 nm-LA (**B**), 117 nm-SA (**C**), and 117 nm-LA (**D**). Data are expressed as means ± SD from three independent experiments with six replicates each. *p* < 0.05 (*) and *p* < 0.01 (**) represent significant difference compared to control (One-way ANOVA followed by Dunnett’s multiple comparison test).

**Figure 4:**
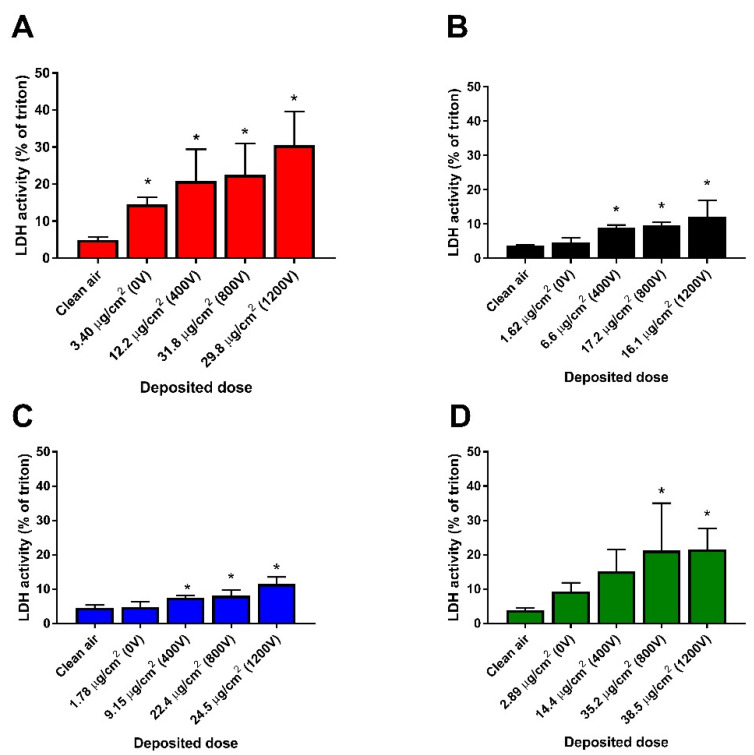
LDH activity measured in HBE cell supernatants after 4 h exposure to TiO_2_ agglomerates at the ALI. 17 nm-SA (**A**), 17 nm-LA (**B**), 117 nm-SA (**C**), and 117 nm-LA (**D**). Data are expressed as means ± SD from three independent experiments with six replicates each. *p* < 0.05 (*) represent significant difference compared to control (One-way ANOVA followed by Dunnett’s multiple comparison test).

After 4 h exposure to aerosolized TiO_2_ agglomerates at the ALI, we measured the effect on cell metabolic activity using the WST-1 assay (Figure 3). Significant loss of metabolic activity (~20%) was observed in cell cultures exposed to smaller agglomerates of 17 nm sized TiO_2_ (17 nm-SA) at the highest dose deposited ~30 µg/cm^2^ (800 and 1200 V) while their larger counterparts (17 nm-LA) induced significant increase in metabolic activity (~25%) at the lowest (~1.6 µg/cm^2^) and at the highest doses (~16 µg/cm^2^) deposited. Although an increasing trend in the metabolic activity can be seen (Figure 3C,D) compared to their clean air controls, small (117 nm-SA) and large agglomerates (117 nm-LA) of 117 nm sized TiO_2_ did not affect the metabolic activity significantly even at the highest doses deposited (~24 and 38 µg/cm^2^, respectively). Subsequently, we measured the LDH activity in the supernatant (basal media) of the cells exposed at the ALI (Figure 4). Compared to clean air exposed controls, a trend of dose dependent increase in LDH activity was noticed for all TiO_2_ agglomerates. 

### 3.4. Oxidative Stress and DNA Damage

**Figure 5:**
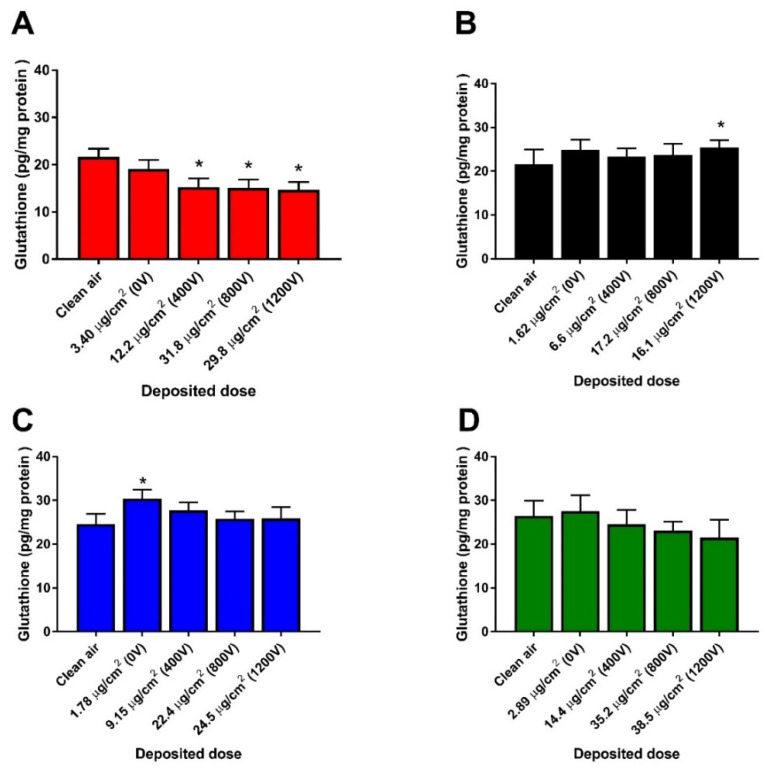
Glutathione levels measured in HBE cells after 4 h exposure to TiO_2_ agglomerates at the ALI. 17 nm-SA (**A**), 17 nm-LA (**B**), 117 nm-SA (**C**), and 117 nm-LA (**D**). Data are expressed as means ± SD from two independent experiments with six replicates each. *p* < 0.05 (*) represent significant difference compared to control (One-way ANOVA followed by Dunnett’s multiple comparison test).

**Figure 6:**
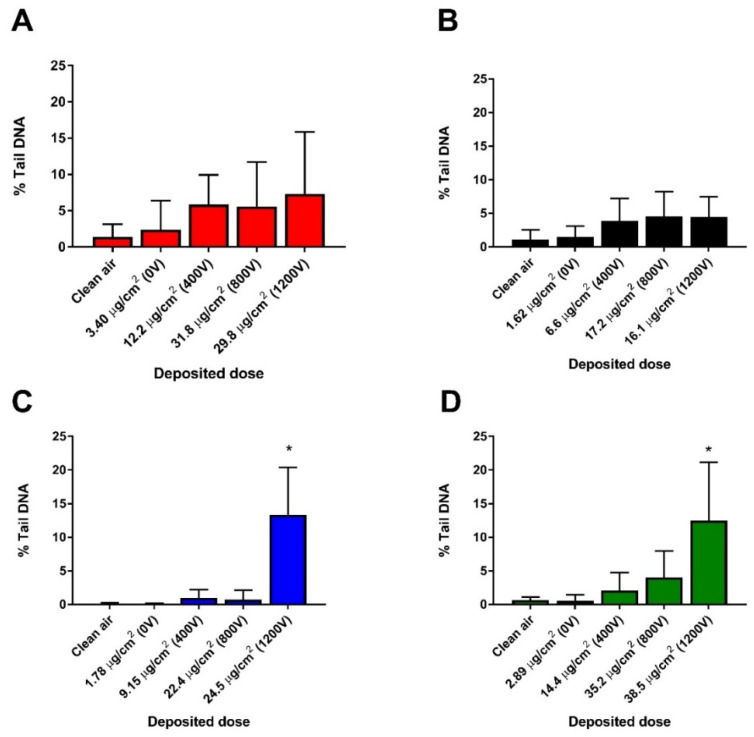
DNA damage measured in HBE cells after 4 h exposure to TiO_2_ agglomerates at the ALI. 17 nm-SA (**A**), 17 nm-LA (**B**), 117 nm-SA (**C**), and 117 nm-LA (**D**). Data are expressed as means ± SD from two independent experiments with six replicates each. *p* < 0.05 (*) represent significant difference compared to control (One-way ANOVA followed by Dunnett’s multiple comparison test).

We measured GSH depletion as an indicator of oxidative stress induction (Figure 5). We detected significant and similar decrease of GSH for 17 nm-SA at doses ~12 and 30 µg/cm^2^ while a slight but statistically significant increase in glutathione was noticed for 17 nm-LA only at the highest dose (~16 µg/cm^2^) deposited. There was a non-significant increase of glutathione for both agglomerates of 117 nm sized TiO_2_ (Figure 5C,D). We assessed the DNA strand breaks as a measure of DNA damage using the alkaline comet assay (Figure 6). An increasing trend in DNA damage was noticed for 17 nm-SA and 17 nm-LA which, however, was not significant compared to unexposed controls (Figure 6A,B). Both SA and LA of 17 nm TiO_2_ NMs induced significant increase in DNA damage only at mass doses ~24 and 38 µg/cm^2^, respectively (Figure 6C,D). 

### 3.5. Summary of Biological Responses

Although there is an overlap of the different deposited doses of the various TiO_2_ agglomerates, for a convenient comparison, we rather considered the significant lowest observed adverse effect concentration for each endpoint to determine the toxic potency of agglomerates (Table 3). Increase in LDH activity and decrease in glutathione was noticed for 17 nm-SA even at low doses (3.4 and 12.2 µg/cm^2^, respectively), which could possibly lead to a decrease in metabolic activity at the higher dose (30 µg/cm^2^), but no such effects were noted for other agglomerates. These results indicate that the smaller agglomerates of nano-sized TiO_2_ are more potent in terms of cytotoxicity and oxidative stress induction at the ALI. However, when considering DNA damage at the different deposited doses, agglomerates of non-nano sized TiO_2_, small agglomerates in particular, appear to be more potent compared to agglomerates of nano-sized TiO_2_. 

Table 4 shows significant lowest observed adverse effect concentrations determined from our previously published study [35] for different endpoints in HBE cells exposed in submerged conditions. None of the agglomerates did induce significant cytotoxic effects at the tested doses but significant decrease in glutathione was noticed for all the agglomerates only at the dose of 155 µg/cm^2^. Large agglomerates of 117 nm-SA induced DNA damage at the dose of 13 µg/cm^2^ while other agglomerates induced such effects at the dose ≥ 26 µg/cm^2^, indicating that the large agglomerates of non-nano sized TiO_2_ are more potent in terms of DNA damage. 

## 4. Discussion

Poor correlation of conventional in vitro and in vivo nanotoxicological exposure studies has been urging the development and validation of models that more closely represent the physiological responses of inhalation exposure. Compared to conventional submerged in vitro systems, air–liquid interface (ALI) exposures are shown to better mimic the inhalation exposure as cell cultures grown at the ALI are exposed to aerosolized particles. However, a deeper understanding of the behavior of NMs in relation to their physicochemical characteristics within the ALI system is essential. In this study, we investigated the influence of TiO_2_ NM agglomeration on their deposition and cytotoxic potency in the ALI system. Our results indicated that dose deposition and their cytotoxic potential are influenced by TiO_2_ agglomeration, particularly for nano-sized TiO_2_.

In the current study, we could deposit in the absence of an EF mass doses of 1.6–3.4 µg/cm^2^, which could be further enhanced in the presence of an EV to 29.8–38.5 µg/cm^2^. Hence, in contrast to submerged exposure where the deposited dose of nano- and non-nano-sized NMs varies drastically, at the ALI similar doses independent of particle size could be deposited as agglomerates. This allows a more direct comparison of dose–response relationships without the need of additional modelling as required under submerged conditions. In a previous study, using the same ALI system, 0.17 µg/cm^2^ and nearly 1.14 µg/cm^2^ was deposited at 0 and 1000 V, respectively, for the same exposure duration using another non-agglomerated nano TiO_2_ (NM-105) [31]. This indicates that the type of TiO_2_ NMs and their agglomeration state influences the dose which is deposited.

We noticed that the smaller agglomerates of nano-sized TiO_2_ NMs induced significant cytotoxicity and oxidative stress at the ALI at low doses (dose < 13 µg/cm^2^) while agglomerates of 17 or 117 nm sized TiO_2_ NMs induced oxidative stress, but no cytotoxicity, under submerged exposure conditions only at the highest dose tested (~155 µg/cm^2^). In the case of DNA damage, small agglomerates of non-nano sized TiO_2_ NMs appear to be more potent at the ALI while large agglomerates of non-nano sized TiO_2_ NMs were found to be the most potent in submerged exposure conditions. These results indicate that the degree of agglomeration influences the potency of TiO_2_ NMs to damage DNA in HBE cells differentially at the ALI and in submerged conditions.

Here, we found that the small agglomerates of nano-sized TiO_2_ NMs (agglomerate size < 100 nm) are more potent in terms of cytotoxicity and oxidative stress induction at the ALI compared to submerged exposure conditions. Noël et al. exposed rats to 7 mg/m^3^ of small (31 nm) and large agglomerates (194 nm) of TiO_2_ NMs for 6h and noticed a significant increase in LDH activity and 8-isoprostane concentration in BALF, which are markers for cytotoxic and oxidative stress effects, respectively [43]. In another study of the same group, rats were exposed to 20 mg/m^3^ of small (29, 28 and 35 nm) and large (156, 128 and 135 nm, respectively) agglomerates obtained from differently sized TiO_2_ NMs (primary size of 5, 20, and 50 nm, respectively) for 6 h [44]. The results indicated that, only the small agglomerates (size < 100 nm) of 5 nm sized TiO_2_ NMs caused a significant increase in cytotoxic effects while the small agglomerates (size < 100 nm) of all TiO_2_ NMs, irrespective of primary particle size, induced a significant increase in oxidative damage compared to larger agglomerates (size > 100 nm), which showed no significant effects for these endpoints. These in vivo results are in agreement with our recent but also previous findings [31], indicating that ALI exposure systems are more suitable than submerged exposure assays to recapitulate adverse effects upon inhalation of NMs. Furthermore, it is of utmost importance to deposit doses in the range of ng to maximally a few ug of nanomaterials per cm^2^ cellular surface area to recapitulate exposure of humans upon inhalation as outlined previously [25].

Recently, the European Food Safety Authority (EFSA) concluded that the use of TiO_2_ as a food additive is no longer considered safe, which highlights the importance to investigate adverse effects of nano-TiO_2_, genotoxic effects in particular [45]. In our previous study, we noticed that the small and large agglomerates of both TiO_2_ NMs used in this study induced DNA damage in HBE cell cultures exposed at submerged conditions at a dose range of <50 µg/cm^2^ (see Table 3) without inducing a significant increase in cytotoxicity and oxidative stress [35]. In this study, both agglomerates of 117 nm TiO_2_ NMs induced DNA damage at the ALI within this dose range also without inducing a significant increase in cytotoxicity and oxidative stress. Our previous study and others have shown that the TiO_2_ NMs were internalized by bronchial epithelial cells in submerged culture [35,46] and such internalized NMs can induce primary DNA damage by directly interacting with DNA, without the induction of cytotoxicity or oxidative stress. In the case of ALI, post exposure incubation for longer periods (such as 24 h) are needed to verify whether the induced DNA damage causes a difference in cell viability or oxidative stress. In contrast, small agglomerates of 17 nm sized TiO_2_ NMs provoked cytotoxicity and oxidative stress but no DNA damage at the same doses. This indicates that genotoxic effects of TiO_2_ NMs are impacted by their agglomeration state, as non-agglomerated TiO_2_ NMs of a modal diameter of 47 nm induced DNA damage already at 1.12 µg/cm^2^ [31]. Moreover, our results further suggest that the genotoxicity of submicron sized TiO_2_ particles or their agglomerates should be also considered in the future.

## 5. Conclusions

In this study, we investigated the influence of agglomeration on the deposition and cytotoxic potency of TiO_2_ NMs at the ALI. Our results indicate that dose deposition and the cytotoxic potential are influenced by agglomeration, particularly for nano-sized TiO_2_ particles. This suggests that the agglomeration state of NMs is crucial in the assessment of pulmonary effects of NMs. Our findings also show that exposure via the ALI induces different cellular responses compared to exposure in submerged systems. More attention should be paid to the methods used to prepare the dispersions of TiO_2_ NMs, specifically concerning agglomeration, in order to assess the (nano) effects at the air-liquid interface and to better predict the hazardous potential of NMs upon inhalation.

## Figures and Tables

**Table 1:** Nomenclature of TiO_2_ agglomerate dispersions.

	17 nm-SA	17 nm-LA	117 nm-SA	117 nm-LA
Particle type	JRCNM10202a	JRCNM10202a	JRCNM10200a	JRCNM10200a
Primary particle diameter	17 nm	17 nm	117 nm	117 nm
Agglomeration state	Small agglomerates	Large agglomerates	Small agglomerates	Large agglomerates

**Table 2:** Characteristics of TiO_2_ aerosols and measured or calculated deposited surface doses on cell cultures.

Material	17 nm-SA	17 nm-LA	117 nm-SA	117 nm-LA
Modal value x_M_ [nm]	72 ± 7	144 ± 12	139 ± 10	71 ± 2
Geometric standard deviation σ_geo_	2.0 ± 0.04	2.0 ± 0.12	2.0 ± 0.1	2.1 ± 0.04
Total number concentration [#/cm³]	4.0 × 10^5^ ± 7.3 × 10^4^	1.1 × 10^5^ ± 3.1 × 10^4^	1.1 × 10^5^ ± 1.5 × 10^4^	4.25 × 10^5^ ± 2.5 × 10^4^
Mass concentration ^a^ c_m_ [mg/m³]	1.6 ± 0.58	1.7 ± 0.45	1.9 ± 0.48	2.0 ± 0.47
Diffusional dose (0 V EF) QCM-signal [µg/cm²]	3.40 ± 0.89	1.62 ± 0.40	1.88 ± 0.62	2.89 ± 0.49
Diffusional dose (0 V EF) TEM analysis [µg/cm²]	n.a. ^b^	1.78 ± 0.73	1.84 ± 0.58	n.a. ^b^
Increased dose (400 V EF) TEM analysis [µg/cm²]	12.2 ^c^	6.6 ± 0.82	9.15 ± 1.44	14.4 ^d^

^a^ calculated from SMPS data, ^b^ n.a. = not analyzed as particles could not be clearly identified by TEM analysis, ^c^ calculated on the basis of QCM data (0 V EF) multiplied with the corresponding factor for enhanced deposition at the different voltages as determined for the 17 nm-LA, ^d^ calculated on the basis of QCM data (µg/cm², 0 V EF) multiplied with the corresponding factor for enhanced deposition at the different voltages as determined for the 117 nm-SA.

**Table 3:** Significant lowest observed adverse effect concentration of different TiO_2_ agglomerates observed for different biological endpoints at the ALI. “-“ indicates no significant effect could be detected.

Dispersions	Highest Dose Deposited (µg/cm^2^)	Decrease in Metabolic Activity (µg/cm^2^)	Increase in LDH Activity (µg/cm^2^)	Decrease in Glutathione (µg/cm^2^)	Increase in DNA Damage (µg/cm^2^)
17nm-SA	30	30	3.4	12.2	-
17nm-LA	16	-	6.5	-	-
117nm-SA	24.5	-	9	-	24.5
117nm-LA	38.5	-	35	-	38.5

**Table 4:** Significant lowest observed adverse effect concentration of different TiO_2_ agglomerates observed for different biological endpoints at the submerged exposure system (from our previously published study). “-” indicates no significant effect could be detected.

Dispersions	Highest Dose Tested (µg/cm²)	Decrease in Metabolic Activity (µg/cm²)	Increase in LDH Activity (µg/cm²)	Decrease in Glutathione (µg/cm²)	Increase in DNA Damage (µg/cm²)
17nm-SA	155	-	-	155	52
17nm-LA	155	-	-	155	26
117nm-SA	155	-	-	155	26
117nm-LA	155	-	-	155	13

## Data Availability

The primary data processed and presented in this study are available on request from the corresponding author.

## Supplementary Materials

The following are available online at https://www.mdpi.com/article/10.3390/nano11123226/s1. Figure S1: Representative TEM micrographs of freshly prepared TiO_2_ stock dispersions, Figure S2: TEM micrographs of aerosolized TiO_2_ agglomerates. Table S1: Characterization of freshly prepared TiO_2_ stock dispersions.
