# Supplementary material for: Agglomeration State of Titanium-Dioxide (TiO2) Nanomaterials Influences the Dose Deposition and Cytotoxic Responses in Human Bronchial Epithelial Cells at the Air-Liquid Interface
Source: Nanomaterials (Basel). 2021 Nov 27;11(12):3226. doi: 10.3390/nano11123226 (PMC8703437; doi:10.3390/nano11123226)
Supplement: Supplementary file 1 [file nanomaterials-11-03226-s001.zip › nanomaterials-1461395-supplementary.pdf]

Supplementary file

Agglomeration state of titanium-dioxide (TiO_2) nanomaterials influences the dose deposition and cytotoxic responses in human bronchial epithelial cells at the air-liquid interface

Sivakumar Murugadoss [1¶], Sonja Mülhopt [2¶*], Silvia Diabaté [3], Manosij Ghosh [1], Hanns-Rudolf Paur [2], Dieter Stapf [2], Carsten Weiss [3#], Peter H. Hoet [1#]

[1] KU Leuven, Environment and Health, Leuven, 3000, Belgium
[2] Institute for Technical Chemistry, Karlsruhe Institute of Technology, Karlsruhe, Germany
[3] Institute of Biological and Chemical Systems - Biological Information Processing, Karlsruhe Institute of Technology, Karlsruhe, Germany
* Correspondence: sonja.muelhopt@kit.edu; Tel.: +49-721-608 23807

[¶ & #] equal contribution

Figure S1 and Table S1 – provided in supplementary as they are published elsewhere [1]

Figure S1: Representative TEM micrographs of freshly prepared TiO_2 stock dispersions of small (SA) and large agglomerates (LA). 17nm-SA (A), 17nm-LA (B), 117nm-SA (C) and 117nm-LA (D).

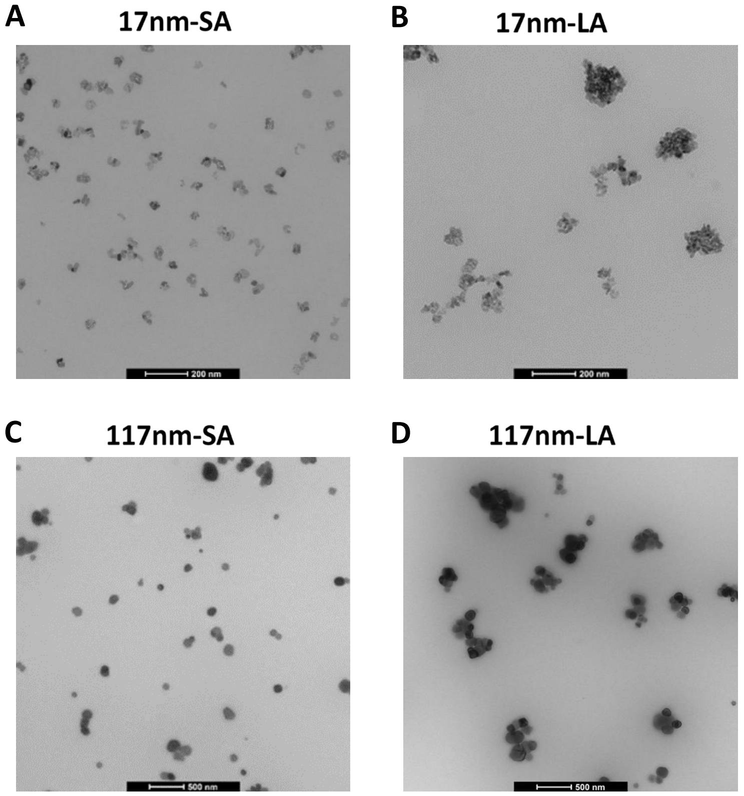

Table S1: Characterization of freshly prepared TiO_2 stock dispersions (2.56 mg/mL).

**Stock suspension**	**Description**	**TEM**			**PTA**	**DLS**	**DLS**
		Median ECD (nm)	Mean ECD (nm)	Mean Feret min (nm)	Mean Hydro-dynamic size (nm)	Z-average (nm)	Zeta potential (mV)
17nm-SA	Small agglomerates of 17 nm sized TiO_2 NP	18	100	33	134	600	33
17nm-LA	Large agglomerates of 17 nm sized TiO_2 NP	127	200	120	207	900	-37
117nm-SA	Large agglomerates of 117 nm sized TiO_2 NP	122	250	148	259	280	-46
117nm-LA	Large agglomerates of 117 nm sized TiO_2 NP	352	500	309	221	580	15

Median and mean equivalent circle diameter (ECD) and mean feret minimum (feret min) measured by transmission electron microscopy (TEM), Z-average (mean hydrodynamic size) by dynamic light scattering (DLS) and mean hydrodynamic size by particle tracking analysis (PTA).

Figure S2: TEM micrographs of aerosolized TiO_2 agglomerates collected over 4h exposure without (0 V) and with (1200 V) electrostatic field (EF) at the ALI exposure system. Magnification 4000 at 80 kV

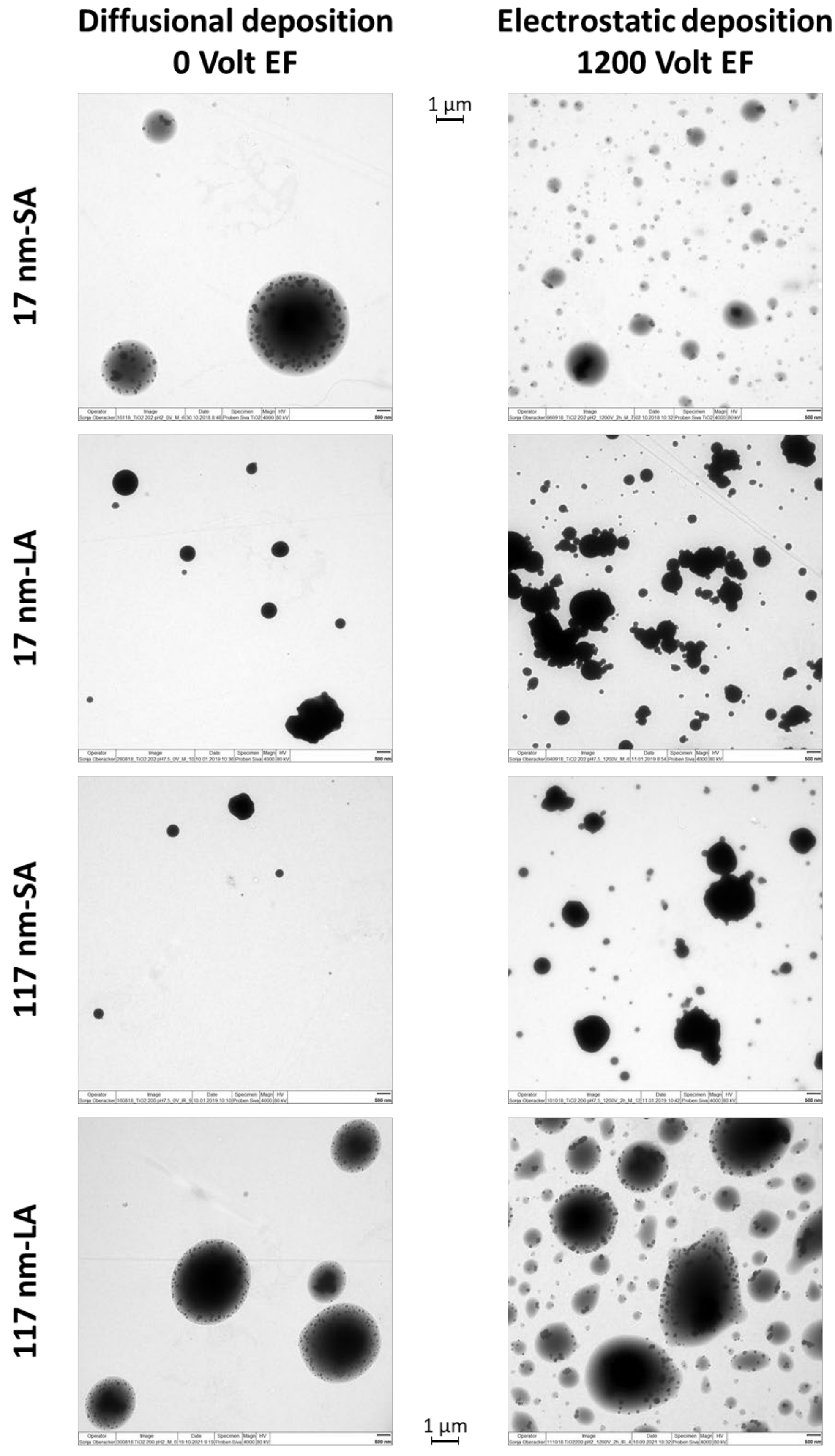

Reference

1. Murugadoss S, Brassinne F, Sebaihi N, Petry J, Cokic SM, Landuyt KL Van, et al. Agglomeration of titanium dioxide nanoparticles increases toxicological responses in vitro and in vivo. Particle and Fibre Toxicology; 2020;7:1–14.
